# Lysenin Channels as Sensors for Ions and Molecules

**DOI:** 10.3390/s20216099

**Published:** 2020-10-27

**Authors:** Andrew Bogard, Gamid Abatchev, Zoe Hutchinson, Jason Ward, Pangaea W. Finn, Fulton McKinney, Daniel Fologea

**Affiliations:** 1 Department of Physics, Boise State University, Boise, ID 83725, USA; andybogard@u.boisestate.edu (A.B.); gamidabatchev@u.boisestate.edu (G.A.); zoehutchinson@u.boisestate.edu (Z.H.); jasonward1@u.boisestate.edu (J.W.); pangaeafinn@u.boisestate.edu (P.W.F.); fultonmckinney@u.boisestate.edu (F.M.); 2 Biomolecular Sciences Graduate Program, Boise State University, Boise, ID 83725, USA

**Keywords:** sensors, lysenin, electrophysiology, translocation, multivalent ions, ligand-gated channels, cationic polymers, gating mechanisms

## Abstract

Lysenin is a pore-forming protein extracted from the earthworm *Eisenia fetida*, which inserts large conductance pores in artificial and natural lipid membranes containing sphingomyelin. Its cytolytic and hemolytic activity is rather indicative of a pore-forming toxin; however, lysenin channels present intricate regulatory features manifested as a reduction in conductance upon exposure to multivalent ions. Lysenin pores also present a large unobstructed channel, which enables the translocation of analytes, such as short DNA and peptide molecules, driven by electrochemical gradients. These important features of lysenin channels provide opportunities for using them as sensors for a large variety of applications. In this respect, this literature review is focused on investigations aimed at the potential use of lysenin channels as analytical tools. The described explorations include interactions with multivalent inorganic and organic cations, analyses on the reversibility of such interactions, insights into the regulation mechanisms of lysenin channels, interactions with purines, stochastic sensing of peptides and DNA molecules, and evidence of molecular translocation. Lysenin channels present themselves as versatile sensing platforms that exploit either intrinsic regulatory features or the changes in ionic currents elicited when molecules thread the conducting pathway, which may be further developed into analytical tools of high specificity and sensitivity or exploited for other scientific biotechnological applications.

## 1. Introduction

The ability of pore-forming proteins and peptides to establish conducting pathways between two sides of a lipid membrane was exploited for decades for numerous analytical applications [1,2,3,4,5,6,7,8,9]. The most common sensing principle relies on measuring changes in the ionic currents elicited by specific and non-specific interactions between analytes of interest and wild-type or engineered protein channels [10,11,12,13,14,15,16,17]. These tiny nano-scale analytical tools present a high electrical gain, hence detection is straightforward with relatively simple amplifiers. Modulation of ionic currents may occur because of selectivity, existence of regulatory mechanisms that lead to conformational changes and conductance adjustments, and diminished ionic flows resulting from analyte binding or translocation through the pore [6,8,15,18,19,20].

Adjustments of conductance in response to chemical stimuli are an essential biological function of canonical ion channels in living cells [18,21,22], and such features may be replicated in vitro for sensing purposes. However, ion channel reconstitution in artificial membrane systems is not always an easy task [23,24,25]. Besides not always being readily available, the channels often have narrow confinements that limit the magnitude of the ionic currents and the size of the analytes passing through. As alternatives, porins and pore-forming toxins present similar functionalities to ion channels in terms of creating transmembrane conducting pathways and ensuring high transport rates [16,26,27,28]. Although they often lack selectivity and regulation, which might be an important characteristic for sensor development, they are amenable to chemical and genetic modifications aiming at introducing specific bio-recognition elements into their structure and changing their response to stimuli [5,13,16,19]. Porins and pore-forming toxins often present a large conducting pathway, which not only ensures greater ionic currents but also allows passage of larger analytes for translocation-based sensing.

Numerous nanopores of biological origin were investigated for sensing applications, such as α-hemolysin, aerolysin, *E. coli* ClyA toxin, lysenin, and motor proteins [5,6,8,29,30,31,32,33,34,35,36]. Among those biological tools, lysenin channels are attractive candidates for sensor development owing to their commercial availability, facile reconstitution into artificial membranes, extended stability, intrinsic regulatory mechanisms, and a large unobstructed opening. Lysenin is a 297-amino-acid pore-forming toxin extracted from the coelomic fluid of the earthworm *E. fetida*, which specifically interacts with sphingomyelin and oligomerizes into large conductance channels in artificial and natural lipid membrane systems [37,38,39,40,41,42,43,44,45,46]. Structural data achieved by employing X-ray crystallography, cryo-EM, and AFM indicate the existence of a large nonameric β-barrel pore (9–11 nm long, and 2–3 nm diameter) and no visible constrictions in the lumen [45,46,47,48,49,50,51]. This large conductance pathway introduced in the cell membranes leads to fast dissipation of the electrochemical gradients responsible for the observed hemolytic and cytolytic activity [39]. Although the toxin may play an important role in the earthworm’s innate immunity and defense strategies [39], the exact physiological role of lysenin has yet to be elucidated. Nonetheless, lysenin channels present a large variety of intricate, sometimes unique features among pore-forming toxins, which make them excellent models for fundamental biological studies and applications. For example, the transitions from soluble form to fully functional transmembrane transporters and the role played by sphingomyelin and cholesterol in membrane binding, oligomerization, and pore formation are extraordinary characteristics of lysenin channels, and they have been addressed in multiple reports and reviews [37,38,41,42,43,44,46,50,52]. In addition, lysenin channels possess some salient features commonly shared by ion channels. Like many ion channels and pore-forming proteins, lysenin has a high transport rate; it also presents a certain selectivity for cations [41], but this is much less apparent compared to the selectivity of many ion channels. What is unusual and uncommon for other pore-forming toxins is the lysenin channel’s regulatory mechanisms. When reconstituted into artificial membrane systems containing anionic lipids, lysenin channels present a strong asymmetrical voltage-induced gating well within the physiological transmembrane voltage range [40,41]. Lysenin channels undergo massive closure at transmembrane voltages exceeding ~20 mV; however, this remarkable feature vanishes when the target membrane is exclusively composed of neutral lipids, in which case lysenin channels remain open for both positive and negative transmembrane voltages [40,41,53]. The voltage-induced gating is influenced not only by membrane composition but also by ionic strength and pH of the support electrolyte [53], which is expected for a gating mechanism that implies interactions between a voltage-domain sensor and electric fields. Although the voltage-induced gating is reversible, return to the open states is realized through a different invariant pathway, leading to a significant hysteresis in conductance [54]. This hysteresis manifests at large time scales, excluding a dynamic origin stemming in the slow activation/inactivation of the channels subjected to oscillatory voltage stimuli.

The requirement for sphingomyelin in the target membrane can be exploited for applications, such as probing lipid rafts [52,55,56,57]. However, two other salient features suggest the potential use of lysenin channels as powerful analytical tools, and these are the major focus of this informative review. Lysenin presents binding sites for multivalent cations and anions; when such compounds are used as analytes, lysenin channels respond by diminishing their conductance proportionally to the concentration of the chemical stimulus [58,59,60,61,62,63]. In most cases, the response is reversible and ligand removal leads to complete restoration of the channel’s conducting properties. The mechanisms by which different chemicals modulate the channel’s conductance depend on the physical properties and chemical identities of analytes, and include simple binding and partial occlusion, conformational changes to closed or sub-conducting states (ligand-induced gating), and gating and trapping of long polymeric molecules [58,59,60,61,62]. In the same line of sensing capabilities, the large opening of lysenin channels and absence of vestibular constrictions recommends them as analytical tools for single molecule detection and characterization by resistive pulse techniques (stochastic sensing) [29,51,58].

Lysenin presents itself with intrinsic sensing capabilities that may be exploited for a large variety of scientific, biomedical, and biotechnological applications. Further channel engineering may lead to development of precise, highly sensitive, and specific sensors with single molecule identification and discrimination capabilities.

## 2. Lysenin Channels as Multivalent Ion Sensors

**Figure A1:**
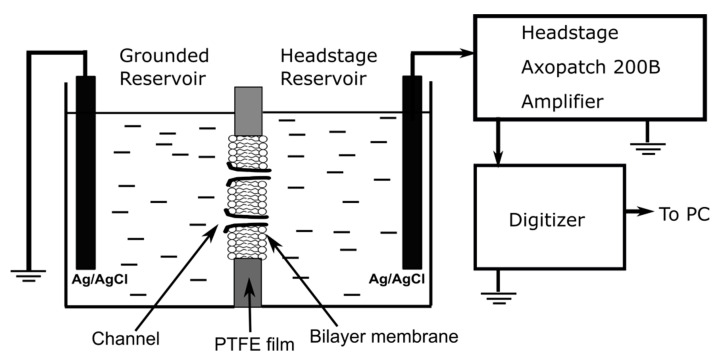
A typical experimental setup for lysenin channel reconstitution into bilayer lipid membranes and electrical measurements of changes in ionic currents induced by interactions with analytes.

A typical experimental setup for assessing the sensing capabilities of lysenin channels by employing electrophysiology approaches is detailed in Appendix A and Figure A1. This setup comprises either reconstitution of large populations of lysenin channels for determination of changes in macroscopic conductance upon interactions with multivalent ions, or single channel analyses that enable identification of regulatory mechanisms responsible for the observed changes in macroscopic currents.

### 2.1. Divalent Metal Cations Modulate the Macroscopic Conductance of Lysenin Channels in a Concentration-Dependent Manner

**Figure 1:**
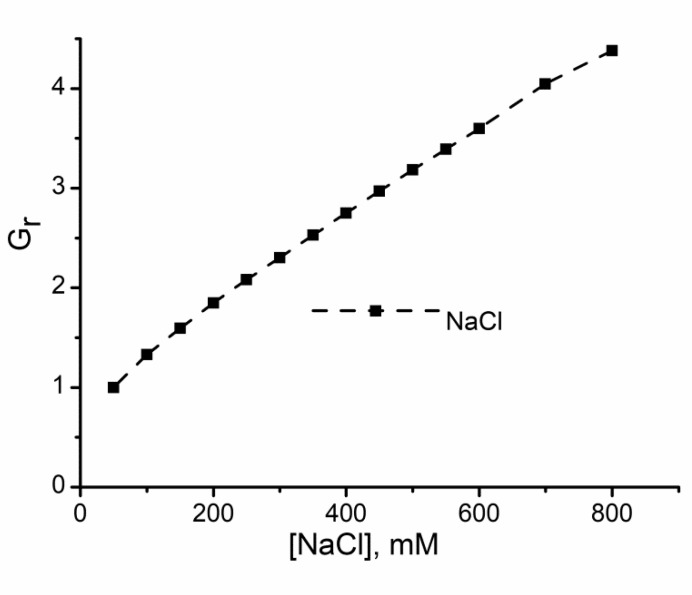
Monovalent ion addition increases the relative conductance G_r_ of lysenin channels by increasing the support electrolyte solution’s conductivity in a concentration-dependent manner. G_r_ = G/G_0_, where G_0_ is the channel’s conductance recorded at the minimal salt concentration (in this case, 50 mM), and G is the channel conductance measured after the addition of ions. The conductance is measured as the slope of I-V plots recorded in the negative voltage range to prevent lysenin gating. Adapted from [62], with permission.

Addition of monovalent ions to the bulk electrolyte solutions bathing lysenin channels inserted into planar lipid membranes leads to an anticipated increase of the relative macroscopic conductance in a concentration-dependent manner (Figure 1) [53,62]. Given the linearity of the plot, there is no doubt that this trend originates in the increased solution conductivity after ion addition; therefore, no change in the channel’s conformation and geometry is observed. Consequently, monovalent anions and cations do not modulate the channel’s conductance other than by adjusting the electrolyte solution’s conductivity [53,62].

**Figure 2:**
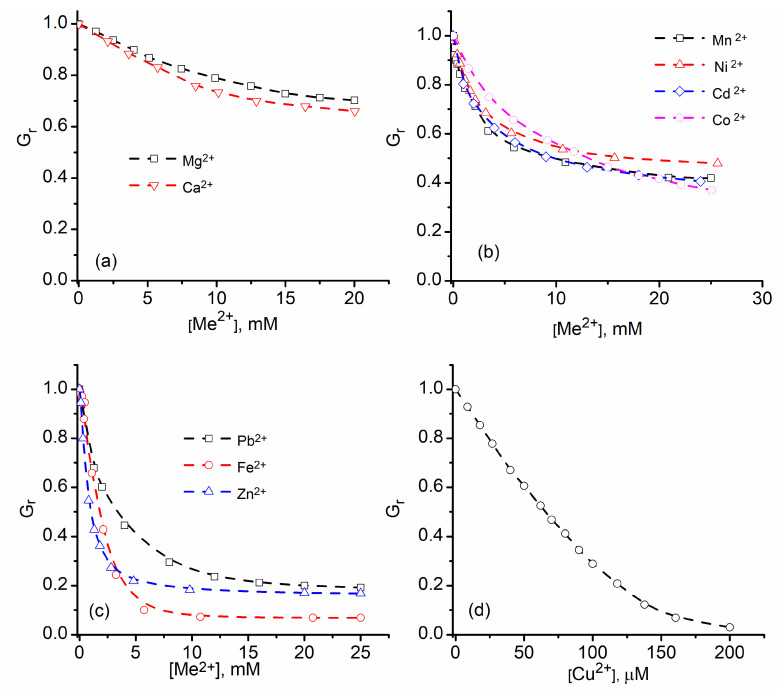
Divalent metal cations inhibit the macroscopic conductance of lysenin channels in a concentration-dependent manner. (**a**) Mg^2+^ and Ca^2+^ addition decreases the macroscopic conductance by ~30%; (**b**) Mn^2+^, Ni^2+^, Cd^2+^, and Co^2+^ inhibit the macroscopic conductance by 50–60%; (**c**) The third group of divalent cations (Pb^2+^, Fe^2+^, and Zn^2+^) shows greater inhibition efficiency; (**d**) Cu^2+^ is the most potent inhibitor among the tested divalent metal ions and practically suppresses the conducting properties at sub-mM concentrations. Adapted from [62] (panels **a**–**c**) and [61] (panel **d**), with permission.

In contrast to monovalent ions, addition of divalent ions elicits a significant decrease of the channel’s conductance [58,61,62]; earlier single channel conductance measurements show that addition of 50 mM CaCl_2_ to the support electrolyte solutions strongly diminishes the ionic currents through lysenin channels [58]. However, this was interpreted as the channel’s charge selectivity against divalent cations. While the channel may present such selectivity, this is not the reason for the reported diminished conductance. Later systematic studies focused on investigating the changes in macroscopic conductance of lysenin channels induced upon addition of increasing concentrations of divalent metal cations showed that the magnitude of the inhibitory effects of divalent metal ions on conductance clearly depends on both electrovalence and chemical identity [61,62]. Increasing amounts of Ca^2+^ and Mg^2+^ ions added to both reservoirs filled with the support electrolyte bathing the channel-containing membrane similarly decrease the macroscopic conductance in a concentration-dependent manner; for both ions, a decrease by ~35% is observed for divalent ion concentration of 20 mM (Figure 2a) [62]. A different group of divalent metals, i.e., Mn^2+^, Ni^2+^, Cd^2+^, and Co^2+^, shows a similar concentration dependency of inhibition but enhanced inhibitory capabilities [61] (Figure 2b). A third group of ions (Pb^2+^, Fe^2+^, and Zn^2+^) diminish the channel’s conductance by a greater extent (~80–90%) when added to the bulk at concentrations up to 25 mM (Figure 2c) [61]. Although the conductance modulation is dependent on the chemical identity of the divalent ions, the inhibition curves are otherwise similar and maximum effects are observed at relatively large concentrations in the bulk (i.e., ~20 mM). A notable exception is Cu^2+^, which is a very potent conductance inhibitor (Figure 2d) and practically cancels the channel’s conducting properties at the 200 µM bulk concentration [61], which is much lower than the ~20 mM required to achieve maximum inhibition for the other divalent ions [61,62].

### 2.2. Trivalent Metal Cations Strongly Inhibit the Macroscopic Conductance of Lysenin Channels

**Figure 3:**
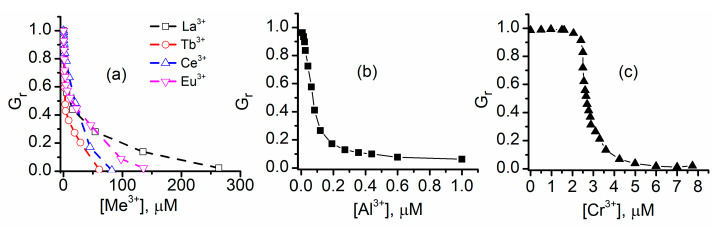
Modulation of the macroscopic conductance of lysenin channels by trivalent metal ions. (**a**) Lanthanide addition completely suppresses the macroscopic conductance in a concentration-dependent manner; (**b**) Al^3+^ ions are strong inhibitors and reduce the conductance to negligible values in the μM range; (**c**) Cr^3+^, a potent conductance inhibitor, presents an inhibition curve that suggests a cooperative process. Adapted from [62] (panel **a**) and [61] (panels **b**,**c**), with permission.

Addition of trivalent metal ions to the support electrolyte solutions also shows a concentration-dependent decrease in the macroscopic conductance of lysenin channels (Figure 3) [61,62]. In contrast to the action of most divalent metal ions, the macroscopic conductance is practically suppressed at trivalent metal ion concentrations in the sub-millimolar range; among all tested divalent metal ions, only Cu^2+^ shows such strong inhibitory capabilities. As with divalent ions, the extent of inhibition depends on the concentration and chemical identity of trivalent ions. The tested lanthanides reduce the macroscopic conductance to negligible near-zero values at concentrations ranging from 50 to 250 µM (Figure 3a), while Al^3+^ shows a much stronger inhibition and produces a similar effect in the µM range (Figure 3b). Nonetheless, the inhibition curves for these trivalent ions are qualitatively similar and resemble the effects recorded for divalent metal ions. A more intricate inhibition curve is presented by Cr^3+^ (Figure 3c), which significantly reduces the ionic transport through the channels at concentrations under 10 µM. However, the concentration dependency of the inhibition is qualitatively different from all the other ions; the pronounced sigmoidal shape suggests a strong positive cooperativity [61], with maximum effects in the range 2–4 µM.

### 2.3. The Changes in Macroscopic Conductance Elicited by Multivalent Cations Are Reversible

**Figure 4:**
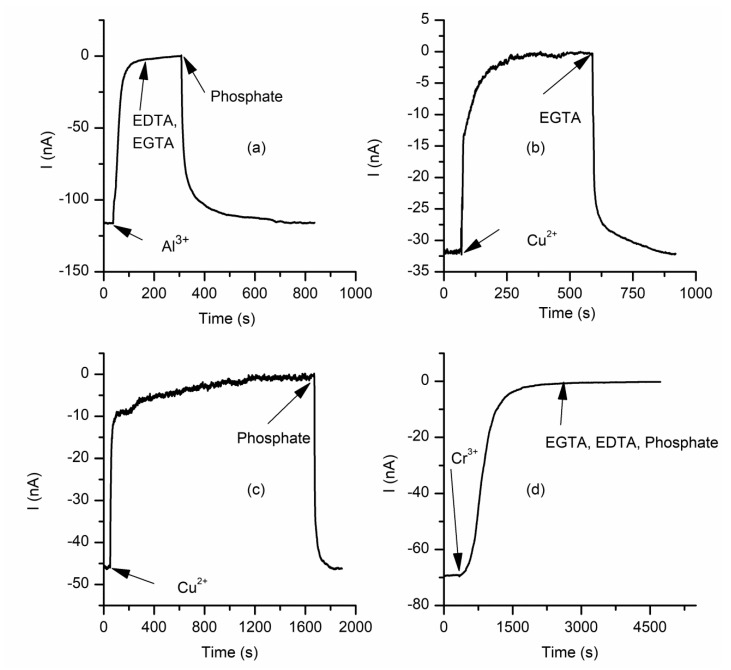
Most, but not all, multivalent metals reversibly interact with lysenin channels. (**a**) Al^3+^ precipitation by phosphate addition reinstates the initial macroscopic conductance. Cu^2+^ removal by EGTA (**b**) or phosphate precipitation (**c**) quickly restores the ionic conductance; (**d**) EDTA, EGTA, or phosphate addition does not cancel the inhibitory effects of Cr^3+^. Adapted from [61], with permission.

The changes in macroscopic conductance of lysenin channels upon exposure to multivalent metal ions may be further exploited for sensing applications. An important feature of such sensors would be their reusability, which is conditioned by the reversibility of interactions with multivalent ions. In this endeavor, a few studies focused on investigating eventual changes in macroscopic conductance manifested upon removal of multivalent ions from the support electrolyte [61,62]. The decrease in macroscopic conductance observed upon addition of small amounts of La^3+^ ions is completely reversed by EDTA addition [62]. EDTA chelates the La^3+^ ions, which reinstates the original macroscopic conductance; therefore, the channel–ion interactions are reversible. This process is fast, which suggests that the multivalent ions likely interact with the inserted channels and adjust their conducting properties rather than damaging or pulling them from the support membrane [62]. Buffer exchange would be the most universal method to remove the multivalent ions from solutions [59], but to avoid membrane rupture during the procedure, chelators and precipitation agents may be used for this task [61,62]. Al^3+^ ions are among the most potent inhibitors of lysenin channels’ conductance but EDTA or EGTA do not chelate them (Figure 4a). However, addition of phosphate ions to the bulk solutions leads to precipitation and fast recovery of macroscopic conductance (Figure 4a) [61]. Cu^2+^ ions, the most powerful divalent inhibitors, may be easily chelated by EGTA (Figure 4b) or precipitated by phosphate (Figure 4c) in a matter of minutes [61], leading to a full restoration in conductance.

Although the interactions between many multivalent metal ions and lysenin channels proved reversible [61,62], Cr^3+^ is a notable exception. Any attempt to chelate or precipitate the Cr^3+^ failed (Figure 4d) [61], but this might be a consequence of the fact that the chemicals used were ineffective as chelators and precipitating agents. However, buffer exchange does not indicate any recovery of the macroscopic conductance even after 12 h [61]; this observation, together with the unique shape of the inhibition curve, indicates that the interactions between Cr^3+^ and lysenin channels are irreversible and realized by mechanisms different from the other multivalent ions.

### 2.4. Lysenin Channels Undergo Ligand-Induced Gating Upon Exposure to Multivalent Cations

**Figure 5:**
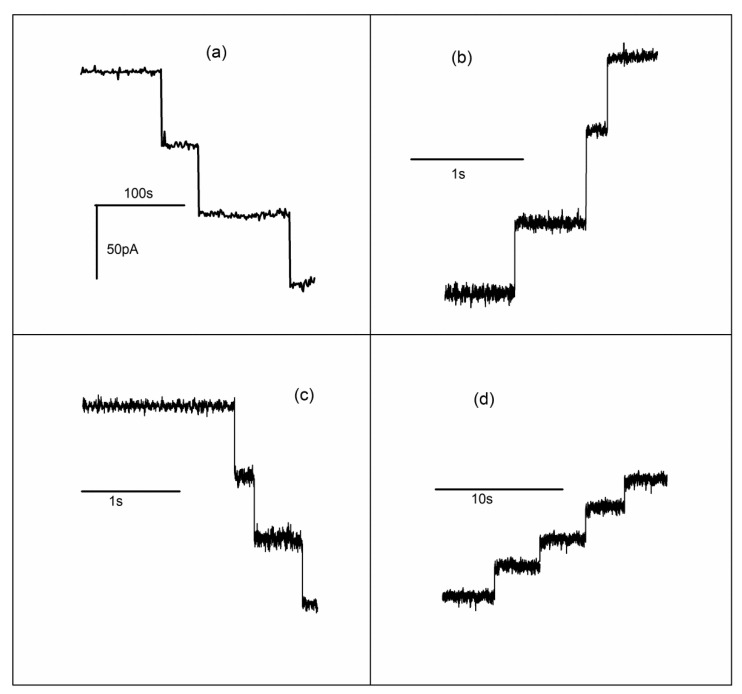
The macroscopic conductance of lysenin channels is adjusted by channel transition to non-conducting or sub-conducting states. (**a**) Insertion of three lysenin channels in the bilayer membrane is indicated by the stepwise variation of the ionic currents; (**b**) La^3+^ addition (final concentration 0.1 mM) induces fast conformational transitions that lead to channel closing; (**c**) EDTA addition (1 mM final concentration) reopens lysenin channels previously closed by interactions with La^3+^ ions; (**d**) Ca^2+^ addition (20 mM final concentration) induces conformational transitions to sub-conducting states. Adapted from [62], with permission.

An important question pertaining to sensing concerns how lysenin channels respond to multivalent ions and adjust their conductance accordingly. In answer to this question, a series of single-channel experiments that monitored the changes in macroscopic conductance upon addition of multivalent metal cations concluded that the major mechanism of interaction is ligand-induced gating triggered by cation binding to a specific binding site present in the channel’s structure [61,62]. After insertion of a few lysenin channels in the target membrane (Figure 5a), La^3+^ addition leads to a stepwise reduction of the single-channel currents (Figure 5b). In terms of ionic currents, the process is simply a reversal of the single channel insertion and the amplitude of the changes in ionic current for each step is identical for the two distinct processes. Addition of EDTA to the support electrolyte again reverses the process but shows an otherwise identical variation of the ionic currents in terms of change/step (Figure 5c). These experiments concluded that the trivalent metal ions induce conformational changes of the channels (gating) from open to fully closed states. Since the conductance of the fully closed channel is negligible, this partially explains the greater inhibition efficiency of trivalent metals by complete cancellation of the macroscopic conductance. Nonetheless, this explanation is not satisfactory for divalent metal ions, for which a flattening of the inhibition curve occurs (see Figure 2) while the macroscopic conductance still has large values. To identify the origin of this behavior, similar single-channel experiments were conducted by employing Ca^2+^ ions as inhibitors [61,62]. As Figure 5d shows, Ca^2+^ addition induces stepwise changes of the ionic currents, which also suggests a gating mechanism. However, the amplitude of each individual variation is roughly half the amplitude corresponding to a fully open channel. This discrepancy was explained by considering that, in contrast to trivalent metals, Ca^2+^ ions trigger conformational transitions from open to partially conducting states [62].

**Figure 6:**
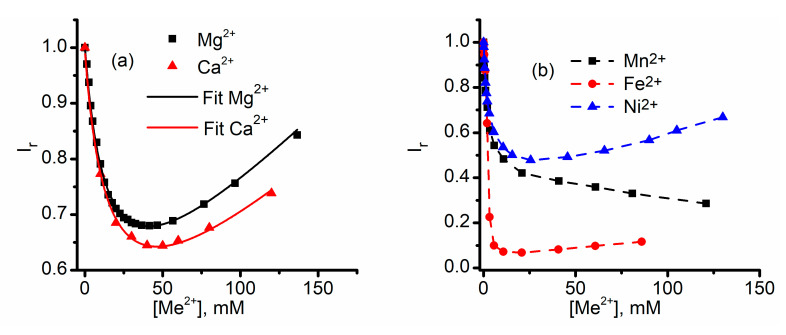
Divalent metal cations induce conformational changes to sub-conducting states. (**a**) The inhibition curves recorded following successive Mg^2+^ and Ca^2+^ additions indicate that the channels are undergoing transitions to sub-conducting states without full closing. The continuous line represents the fit of experimental data with Equation (1); (**b**) The inflection point in the inhibition curves suggests that other divalent cations also induce transitions to stable sub-conducting states. Adapted from [62] (panel **a**) and [61] (panel **b**), with permission.

The single channel recordings performed in the presence of Ca^2+^ ions do not provide sufficient information with regards to channels undergoing single transitions from open to sub-conducting states as opposed to full closing in two or more steps. To detail the mechanism, the interaction with divalent metals was described as a simple Langmuir isothermal absorption process and a formula for the relative changes in macroscopic currents was derived [62]:

(1) $$\frac{I}{I_{t}}=\left(\frac{{(K}_{0}+{b[\mathrm{Me}}^{2+}])}{K_{0}}\right)\left(1-\left(1-f\right)\left(\frac{1}{1+\left(\frac{1}{{\alpha [\mathrm{Me}}^{2+}]}\right)}\right)\right),$$

where *I* is the current through the fully open channels, *I_t_* is the current after *Me^2+^* addition (both currents are measured at the same voltage, therefore their ratio represents the relative change in macroscopic conductance G_r_), *K*_0_ is the specific conductivity of the bulk before *Me*^2+^ addition, *b* is a factor accounting for the linear changes in conductivity upon *Me*^2+^ addition, *f* is the ratio between the open/sub-conducting channel conductance in otherwise identical conditions, and α is the equilibrium constant of the channel–ion binding process [62]. The above equation predicts that for channels undergoing transitions to only sub-conducting states (no full closing, irrespective of the inhibitor’s concentration), the currents should first decrease until all channels attain sub-conductance, after which the currents should increase upon ionic additions owing to the increased conductivity of the solution. In contrast, a full closing of the channel in two or more steps would lead to a continual decrease of the ionic currents in response to an increasing inhibitor concentration. This model was tested for investigating the macroscopic currents recorded in the presence of Ca^2+^ and Mg^2+^, and the excellent fit of experimental data with Equation (1) (Figure 6a) demonstrate the existence of highly stable sub-conducting states upon influence exerted by the divalent cations. In the same line, stable sub-conducting states were also suggested for other divalent metal ions, as inferred from local minima in the inhibition curves (Figure 6b) [61]. The different inhibitory effects may be explained by accounting for more than one sub-conducting state or assuming that not all the ions lead to the same conductance ratio between the open and sub-conducting states.

**Figure 7:**
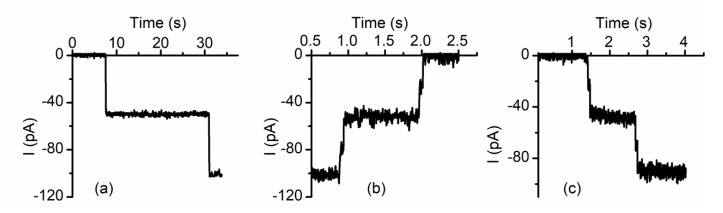
Cu^2+^ induces full closing of lysenin channels through intermediate steps. (**a**) The insertion of two lysenin channels into a planar membrane is indicated by the stepwise variation of the ionic current; (**b**) Cu^2+^ addition (500 µM) induces full closure of the channels, but each closure comprises two steps; (**c**) Complete channel reopening upon Cu^2+^ removal by addition of EGTA (10 mM) is also realized through intermediate sub-conducting states. Adapted from [61], with permission.

Cu^2+^ ions show a different behavior that does not match the typical description of divalent ion effects in terms of inhibition efficiency and the shape of the inhibition curve (does not present an inflection point), hence resembling trivalent-like effects. To identify the origin of this behavior, single-channel experiments that employed Cu^2+^ ions as inhibitors were conducted similarly to the other divalent and trivalent metal ions. After insertion of only two lysenin channels in the bilayer membrane (Figure 7a), Cu^2+^ addition (500 µM final concentration) completely cancels the individual conductance and reduces the ionic currents to zero in a stepwise manner [61]. However, the transition from open to close is not direct and comprises a short-lived intermediate sub-conducting state (Figure 7b). EGTA addition fully restores the initial conductance of each channel, but the close–open transition is also realized through short intermediate sub-conducting states [61] (Figure 7c). Therefore, Cu^2+^ induces transitions to sub-conducting states, as observed for other divalent metals, but the sub-conducting states are not stable and the channels may fully close by employing a second transition from the sub-conducting to fully closed states [61].

### 2.5. Which One Matters, Charge, or Size?

**Figure 8:**
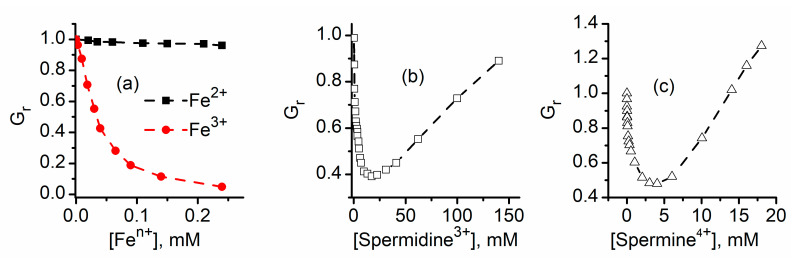
Charge and size influence on the inhibitory effects of multivalent cations. (**a**) The inhibitory effects presented by Fe strongly depend on the ionic charge, and Fe^3+^ is a more efficient inhibitor than Fe^2+^. Voluminous organic ions, such as spermidine^3+^ (**b**) or spermine^4+^ (**c**), present less inhibitory efficiency, in spite of their large charge. Additionally, the inflection point in the inhibition curves indicates that both cations modulate the channel’s conductance by inducing transitions to sub-conducting states. Adapted from [62] (panels **a**,**b**) and [61] (panel **c**), with permission.

With a few exceptions, conductance inhibition is more potent for trivalent than divalent metal cations. The macroscopic currents decrease by a much larger extent for trivalent metal ions, and this may be partially explained by their ability to induce conformational changes that lead to complete channel closure. Nonetheless, the concentration required to achieve ligand-induced gating (full closing, or transitions to sub-conducting states) is much smaller for trivalent metals (in the µM range) than for divalent metals (mM range). This naturally leads to the hypothesis that the charge of the cations is central for the ligand-gating mechanism manifested in the presence of multivalent metal ions. This may be easily seen in Figure 8a, in which the inhibitory effects of Fe ions are more prominent for Fe^3+^ than Fe^2+^ [62]. To better understand the role played by the charge in the gating mechanism, the investigations employed the use of larger organic multivalent ions, such as spermidine^3+^ and spermine^4+^ [61,62]. The inhibition curves for the two voluminous ions (Figure 8b,c) reveal that both ions, despite bearing large charges, exhibit inhibition curves resembling the lysenin channel behavior observed upon exposure to divalent ions. Apart from the necessity of using relatively large cation concentrations to achieve conductance inhibition (in the mM range), the inflection point in the inhibition plots suggests that the gating mechanism implies transitions to sub-conducting states. Therefore, both charge and size (or in other words charge density) play a major role in establishing the channel’s sensitivity to ions and modulating its transition to closed or sub-conducting states.

### 2.6. Cationic Polymers Irreversibly Block Lysenin Channels

**Figure 9:**
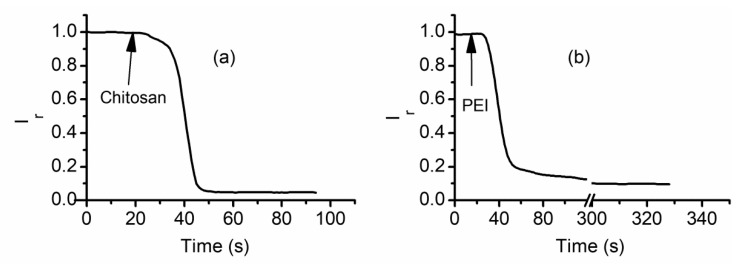
Cationic polymers inhibit the ionic currents through lysenin channels. The evolution of the relative ionic currents measured through lysenin channels upon exposure to (**a**) 8 µM chitosan, and (**b**) 4 µM PEI. Published under Creative Common Attribution License in [63].

Experimentations with multivalent inorganic and organic cations revealed conductance inhibitory effects dependent on both the charge and size of used ions. Conductance modulation is in most cases reversible, and inhibitor removal by chelation, precipitation, or buffer exchange restores the lysenin channel’s conducting properties. However, the inorganic and organic ions used for these investigations were still small compared to the channel’s opening and carried a relatively small charge. This led to questioning of the potential effects on the macroscopic conductance of lysenin channels presented by large and highly charged molecules, such as cationic polymers [63]. In this line of inquiries, the effects on macroscopic conductance of two polyions (i.e., polyethyleneimine (PEI) and chitosan) were evaluated [63]. Both polymers reduce the transport capabilities when added to the bulk electrolyte in the low concentration range (Figure 9), demonstrating strong inhibitory capabilities. However, a major difference was encountered with respect to reversibility: buffer exchange does not reveal any recovery of the conducting properties even after extended exposure to polymer-free electrolyte solutions [63].

**Figure 10:**
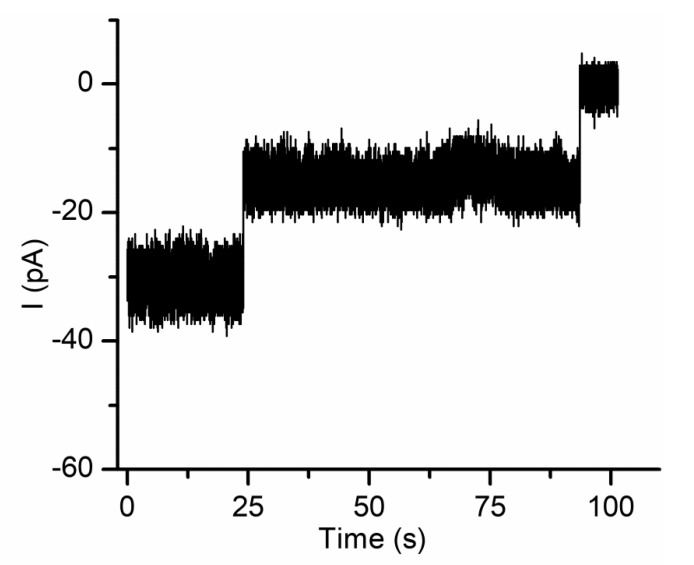
Cationic polymers inhibit the ionic currents in a stepwise manner. PEI addition (10 µM final concentration) rapidly closes the two lysenin channels inserted into the bilayer lipid membrane. Published under Creative Commons Attribution License in [63].

This lack of reversibility is explained by considering a channel occlusion mechanism based on gating and trapping [63]. Once the long polymer enters the channel’s lumen, the large positive charge induces transitions (gating) to either closed or sub-conducting states; this transition happens before the long polymer exits the pore, therefore the polymer is trapped inside the channel and the numerous positive charges present on the chain prevents reopening. This hypothesis is supported by experiments performed on single lysenin channels exposed to cationic polymers [63]. As Figure 10 shows, the two inserted channels undergo a stepwise variation of the open currents upon chitosan addition, which suggests a complete blockage of the conductance pathway. Such complete blockage may also be achieved even if the channels transition to a sub-conducting state but the polymer molecules are trapped within.

### 2.7. Ligand and Voltage Gating of Lysenin Channels Are Not Coupled

**Figure 11:**
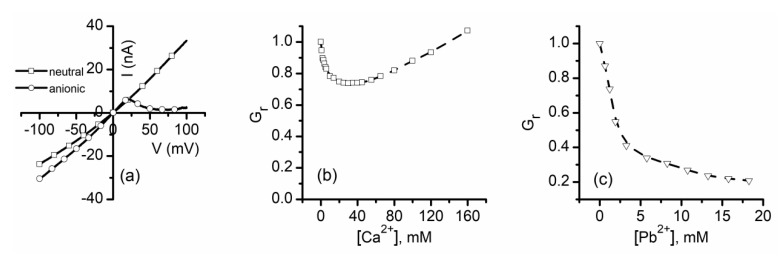
Voltage and ligand-induced gating are realized through distinct mechanisms. (**a**) Lysenin channels inserted into a membrane containing anionic lipids shows the voltage induced gating; a membrane support composed of neutral lipids suppresses voltage regulation and leads to a straight I-V curve. A neutral membrane does not cancel the conductance inhibition presented by Ca^2+^ (**b**) or Pb^2+^ (**c**). Adapted from [61], with permission.

A large body of evidence supports the hypothesis that lysenin channels exposed to multivalent cations transition to non-conducting or sub-conducting states by mechanisms characteristic to ligand-induced gating. However, lysenin channels also present a strong voltage-induced gating, which manifest as reversible complete channel closure at positive transmembrane voltages [40,41]. This regulatory mechanism raises the question whether the two gating mechanisms (i.e., voltage and ligand induced) are related. To address this fundamental question, the inhibitory effects of metal cations were evaluated in experiments that used neutral lipids to produce the lipid membrane, which suppressed the lysenin channel’s voltage-induced gating [40,41]. Upon insertion into membranes containing anionic lipids, lysenin channels show a strong voltage-induced gating, while the use of electrically neutral lipids abrogates this remarkable regulatory feature and leads to a linear I-V plot (Figure 11a). In spite of changes in voltage-gating regulation, lysenin channels inserted into neutral membranes do not show changes in their sensitivity to ions [61]: Ca^2+^ (Figure 11b) and Pb^2+^ (Figure 11c) additions inhibit the macroscopic currents and the inhibition curve presents the inflection point characteristic of stable sub-conducting states. In the same line, irreversible changes in the lysenin channel’s conductance induced by cationic polymers when neutral membranes are used [63] support the hypothesis that voltage and ligand-induced gating are realized through distinct mechanisms.

### 2.8. Cationic Ions and Polymers May Compete for the Binding Sites

**Figure 12:**
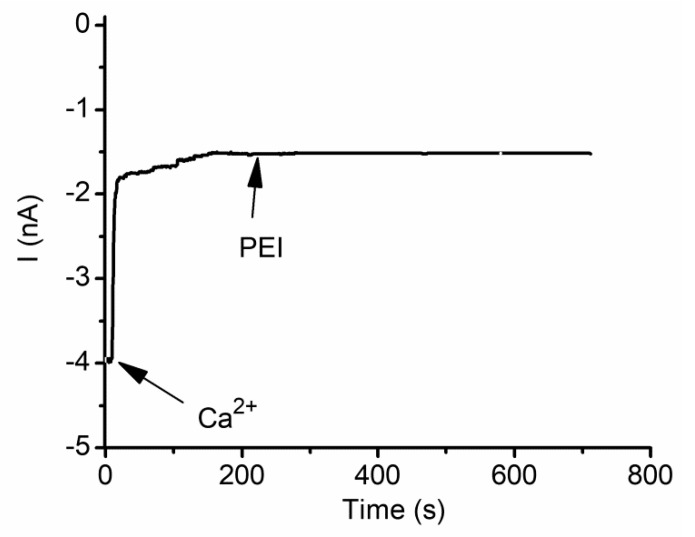
Multivalent ions and cationic polymers compete for the binding sites present in the lysenin channel’s structure. After channel blockage and transition to sub-conducting states by Ca^2+^ addition (40 mM final concentration), PEI (10 µM final concentration) does not show further inhibition of the ionic currents. Published under Creative Commons Attribution License in [63].

The gating and gating/trapping mechanisms are different but may be triggered by similar electrostatic interactions between charges and binding sites present in the channel’s structure. Are these binding sites the same? To answer this question, the investigations focused on assessing a potential competition for occupancy between divalent metal cations and cationic polymers [61]. The results presented in Figure 12 show that the inhibitory effects of PEI are canceled if the channels were previously exposed to large amounts of Ca^2+^ ions. While this suggests that the two inhibitors may compete for the same binding sites, it is also possible that the channel’s transition to sub-conducting states may prevent the polymer’s access to the lumen and further trapping.

### 2.9. Lysenin Interactions with Purines

**Figure 13:**
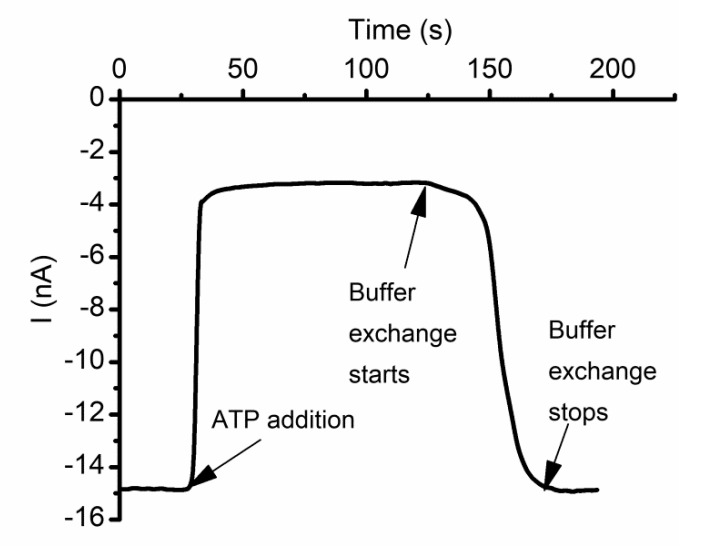
ATP reversibly modulates the macroscopic ionic currents through lysenin channels. The open current through lysenin channels undergoes a major decrease after ATP addition (10 mM final concentration). Buffer exchange with ATP-free support electrolyte reinstates the original conductance, indicative of reversibility. Adapted from [59], with permission.

All the experiments conducted on inorganic and organic ions did not reveal any influence on the channel’s conductance presented by small inorganic anions, which apparently do not interact with lysenin. However, a significant conductance modulation is observed when purines (ATP, ADP, and AMP) are added to the support electrolyte [59]. Addition of ATP (20 mM final concentration) to the support electrolyte quickly reduces the macroscopic ionic currents established through lysenin channels (Figure 13). However, buffer exchange with ATP-free solutions fully reinstates the conducting properties and proves reversibility. Single-channel experiments show that the interactions between lysenin channels and ATP do not imply gating [59], and suggest binding and partial occlusion as plausible explanations.

**Figure 14:**
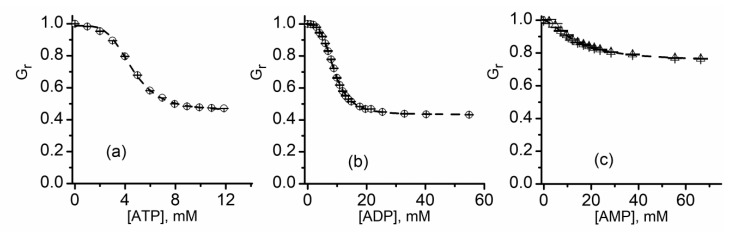
Changes in relative conductance induced by addition of ATP, ADP, or AMP. The relative changes in macroscopic conductance G_r_ show that ATP (**a**) and ADP (**b**) were more efficient inhibitors compared to AMP (**c**). The dashed lines in each panel represent the fit with the Hill equation, which is used to determine *IC*_50_ and *n*. Adapted from [59], with permission.

Figure 14 shows that the relative macroscopic conductance of lysenin channels decreases upon ATP, ADP, or AMP addition in a concentration-dependent manner. The inhibitory effects manifest in the mM range for all three purines, but their potency decreases in the order ATP > ADP > AMP. The shape of the inhibition plots observed for interactions with purines are slightly different than the typical parabola shape recorded for most divalent and trivalent cations and suggest a cooperative process. To better understand the effects of purine inhibitors in relation to cooperativity, a fit of the experimental data was performed by employing the Hill equation [59]:

(2) $$G_{r}=1-(1-G_{\min})\frac{{\left[x\right]}^{n}}{{\left[IC_{50}\right]}^{n}+{\left[x\right]}^{n}},$$

where *G_r_* is the relative macroscopic conductance, *G*_min_ is the minimum relative conductance measured at saturation (all potential binding sites are occupied), *IC*_50_ is the half-way inhibitory concentration, *x* is the purine concentration, and *n* is the Hill coefficient.

The results shown in Table 1 indicate that *IC*_50_ and *n* vary with the chemical identity of the inhibitor; *IC*_50_ increases (lower binding affinity) and *n* decreases (less cooperativity) as the net charge of the anion increases. This suggests that the inhibitory mechanism relies on electrostatic binding of purines to specific sites present in the channel’s lumen [59], which are different from the binding sites implied in cation-induced ligand gating.

**Figure 15:**
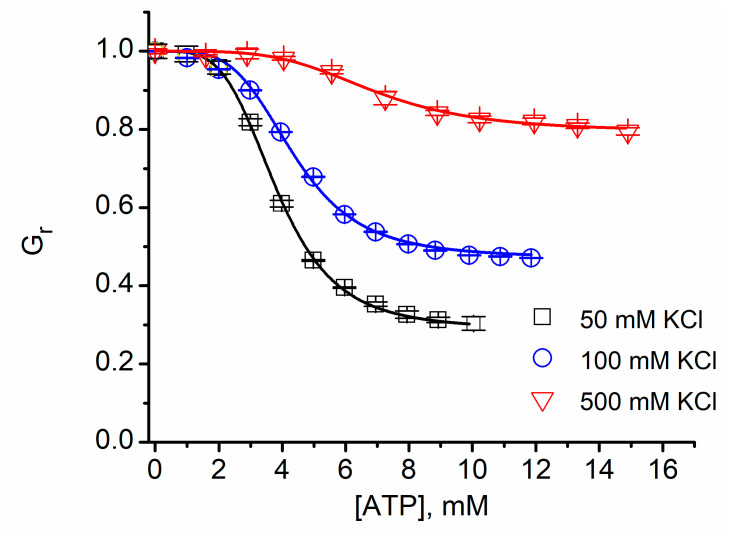
Ionic screening reduces ATP inhibitory effects. The relative conductance indicates that increased ionic screening elicited by addition of KCl minimize the conductance changes induced by ATP addition. The continuous lines represent the fit with the Hill equation (Equation (2)). Adapted from [59], with permission.

The electrostatic nature of the interactions was confirmed in experiments that investigated the effects of ionic screening on ATP-induced inhibition; indeed, the inhibitory effects reported upon electrostatic screening (Figure 15) significantly depend on the ionic strength of the support electrolyte, and *IC*_50_ decreases as the ionic strength increases (Table 2). However, irrespective of the ionic strength, the Hill coefficient *n* does not significantly deviate between experiments (Table 2), hence providing a framework for including effects of molecular identity and structure to explain the differences in the binding affinity of purines [59].

## 3. Lysenin Channels as Stochastic Sensors: Translocation of Macromolecules

Since the pioneer work carried by Kasianowicz et al. showing that biological nanopores may be exploited as tools for single molecule detection and characterization [64], the field of nanopore-based technology has developed at an unprecedented pace. The principle of sensing, a direct expansion of the long-revered Coulter measuring method at the nano-scale [65], is deceptively simple: the resistive pulse technique relies on recording the changes of the ionic currents established through a nanopore when single molecules are electrophoretically driven through the conducting pathway. This research field was initiated by using α-hemolysin as a prototype pore; however, scientists developed and utilized a large variety of synthetic and biological nanopores for similar purposes [6,8,14,33,34,66,67,68,69,70,71,72,73,74,75,76,77,78,79]. The great interest in this topic is fueled by the promise of fast and reliable sequencing of nucleic acids and peptides [14,35,73,74,76,80], development of sensors for single molecule detection and characterization, fast and reliable determination of biomolecules in complex biological samples, and many other analytical applications. The use of lysenin channels as resistive-pulse sensors may present some clear advantages over synthetic and natural nanopores: the channel’s opening is relatively large and therefore able to accommodate large analytes, single and multiple channels may be easily reconstituted into membranes, and the inserted channels are very stable. The voltage-induced gating that manifests at positive bias potentials may be considered an impediment for some of the applications, but it may be easily suppressed by using neutral lipids to create the support lipid membrane.

### 3.1. DNA Translocation Experiments

In spite of its potential, reports on lysenin use as a stochastic sensor are scarce. Single-channel experiments developed by Aoki et al. [58] show that spikes in the open current occur when DNA is added to the bulk electrolyte solution. However, the focus of those investigations was different, and it is not clear if the recorded transients in the ionic current are indicative of DNA molecule translocation. Another report focused on investigating the lysenin channel’s structure shows some preliminary investigations on DNA translocation [51]. The exploration indicates that wild-type lysenin channels are not able to support DNA translocation, most probably owing to the strong repulsion between charged polymers and charged domains in the channel’s structure; in contrast, a mutant version constructed by replacing negatively charge amino acids with neutral and cationic ones shows transient changes in the ionic current, resembling translocation [51]. While the results indicate that the wild-type channel and the engineered one have different properties with regards to translocation, more experimental evidence should be provided in support of the claim that lysenin successfully captures and facilitates translocation of DNA strands. The transient signal was obtained by using a mixture of aptamer DNA and its target molecule (thrombin), hence the source of the variation of the ionic currents is uncertain.

**Figure 16:**
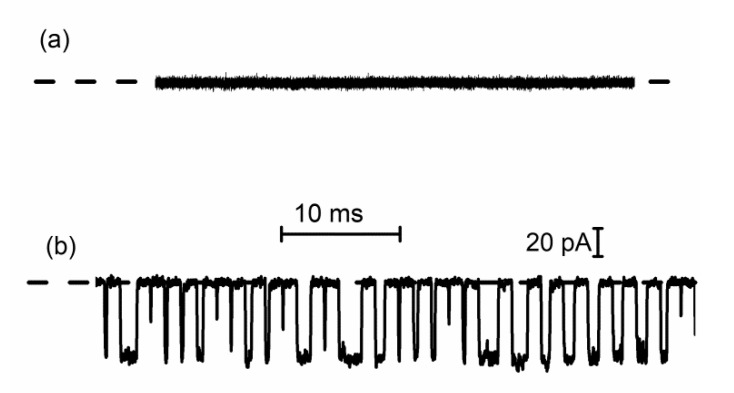
DNA translocation through single lysenin channels. (**a**) ssDNA addition to the headstage wired reservoir biased by a negative potential does not elicit transient changes in the open current through a single lysenin channel, indicative of absence of translocation; (**b**) ssDNA addition to the opposite side (grounded reservoir) and application of a positive voltage to the headstage-wired reservoir leads to events resembling translocation.

Therefore, it is worth mentioning and presenting investigations performed by our group on DNA translocation through lysenin channels [81]. To suppress the voltage-induced gating that manifests at positive voltages, single lysenin channels were reconstituted into a bilayer lipid membrane composed of neutral lipids [40,41]. No transient changes in the open ionic current were visible when 69 nt DNA (5 nM final concentration) was added to the reservoir wired to the headstage and biased by a negative potential (Figure 16a). Since the electric field for this configuration has the correct orientation to drive the DNA molecules through the channels into the opposite reservoir, the logical conclusion is that the DNA molecules do not thread the channels. However, polarity reversal (positive potential on the reservoir connected to the headstage) and ssDNA addition to the grounded reservoir elicits fast and deep changes in the open ionic current of a single lysenin channel, resembling translocation (Figure 16b). These observations confirm that wild-type lysenin may prevent DNA translocation (most probably due to electrostatic repulsions) when the molecules are added to the stem side of the channel. They also confirm the necessity to suppress the voltage-induced gating by using neutral lipids for ssDNA translocation experiments.

**Figure 17:**
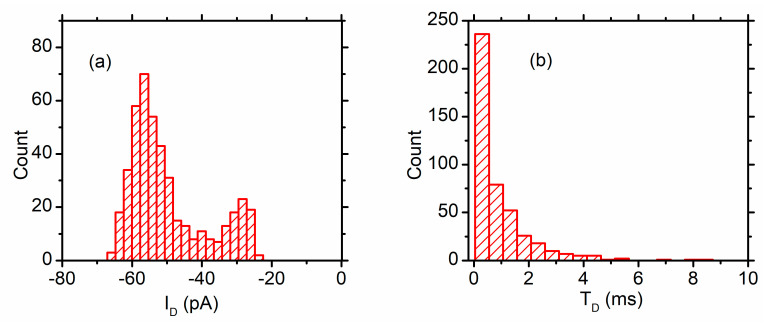
Analysis of translocation events. (**a**) The distribution of the current blockages indicates two peaks centered at ~55 and ~28 pA, respectively. The low amplitude peak may originate in ssDNA channel collisions, while the high amplitude peak represents putative translocations; (**b**) The dwell time of the events follows an exponential decay, characteristic to translocation through “sticky” pores.

From Figure 15 and the distribution of the current blockage (I_D_) and dwell time (T_d_) (Figure 16), one may easily observe that the current drops are relatively uniform for the recorded events (~ 56 pA); however, some short and reduced-magnitude spikes are observed in both the current trace (Figure 16b) and I_D_ histogram (Figure 17a). This type of noise is common in translocation experiments, and it is considered a consequence of molecules colliding with the mouth of the pore without being captured by the electric field and translocated [70,80,82,83]. As opposed to the relatively uniform changes in the ionic current, the dwell time seems to be not only unusually long for some events but also extremely variable compared with other ssDNA translocation experiments (Figure 17b). This might be a consequence of DNA “stickiness” to the channel’s lumen, which may be explained based on the investigations of lysenin channel interactions with purines (vide supra, and [59]). The resulting exponential decay shape of the dwell time distribution is common for macromolecule translocation through narrow “sticky” nanopores [34,80,82,84,85,86].

**Figure 18:**
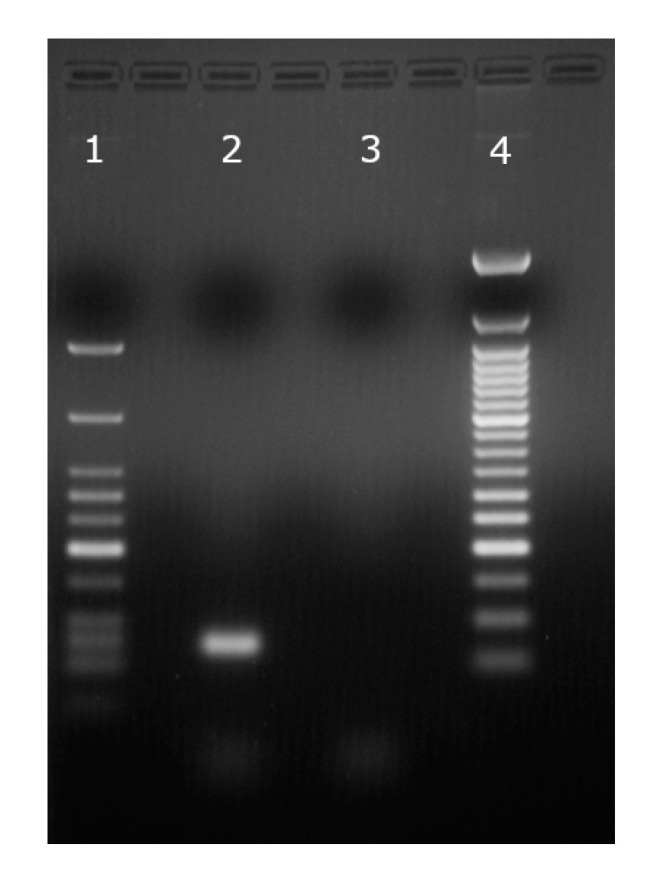
The analysis of translocated ssDNA performed by gel electrophoresis (2% agarose) after PCR amplification. (1) Low Molecular Weight Marker (25–766 bp), New England Biolab; (2) Amplicon produced from ssDNA molecules translocated through lysenin channels; (3) The absence of DNA indicates that a reversed polarity prevents translocation. (4) 50 bp Ladder Marker, New England Biolabs.

Protein channel gating in the presence of DNA may lead to “events” that resemble translocation and additional evidence is needed to demonstrate DNA passage. Irrespective of the origin of the differences between events, PCR provides irrefutable evidence of DNA translocation [64]. In this case, DNA amplification by PCR after solution extraction from the reservoirs and further analysis shows that the DNA translocation process was successful. The gel electrophoresis analysis (Figure 18) of the PCR-amplified sequence in the presence of forward and reverse primers shows the presence of translocated DNA molecules, and the two markers aid identification by molecular weight. In addition, the sample collected from identical experiments but for which the voltage was reversed show no detectable amplicon in the reservoir, indicating that the current blockages represent DNA passage through lysenin channels.

### 3.2. Peptide Translocation

**Figure 19:**
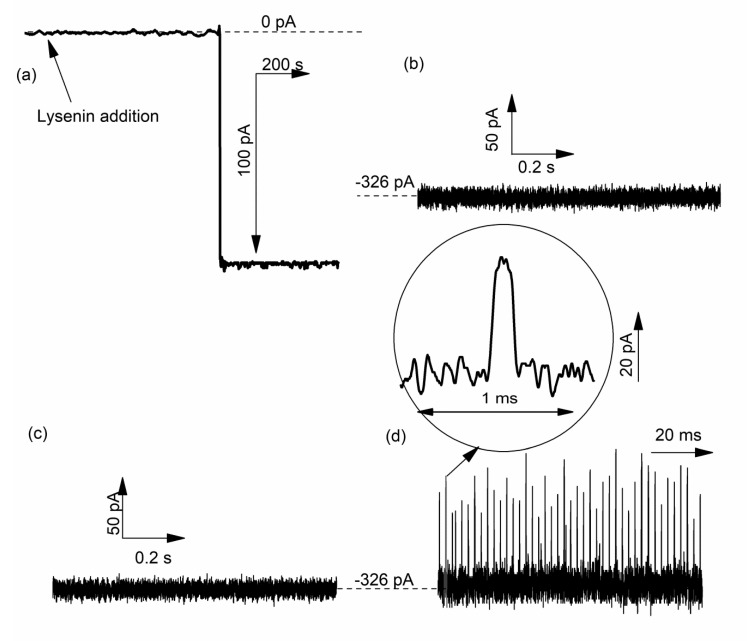
Ang II peptide translocation through single lysenin channels. (**a**) The insertion of single channels was monitored at −60 mV transmembrane potential. No changes in the open current established through two lysenin channels at −80 mV is observed when: (**b**) no Ang II is added, and (**c**) Ang II is added to the reservoir held at negative potential; (**d**) Ang II addition to the positively-biased reservoir elicits transient changes that resemble translocation. The inset shows a single translocation event. Published under Creative Commons Attribution License in [29].

The large interest in DNA translocation was fueled by the promise of fast and reliable sequencing [64,66,87,88]. However, single molecule detection and characterization of peptides molecules is equally important; numerous synthetic and natural nanopore sensing platforms have been employed for such tasks [70,82,84,85,89,90], and lysenin is one of them [29]. Lysenin was investigated as a stochastic sensor for the short octameric peptide angiotensin II (Ang II) [29]. After single channel reconstitution in neutral lipid bilayers (Figure 19a), no transient changes in the ionic current established through two channels was observed at −80 mV bias potential (Figure 19b). A similarly quiet baseline was recorded after Ang II addition to the reservoir hardwired to the headstage (Figure 19c); Ang II is a positively charged peptide, and the particular orientation of the electric field prevented its translocation. However, peptide addition to the ground reservoir in otherwise identical electrical and solution conditions shows frequent transient changes in the open ionic current, resembling translocation through other biological channels (Figure 19d).

**Figure 20:**
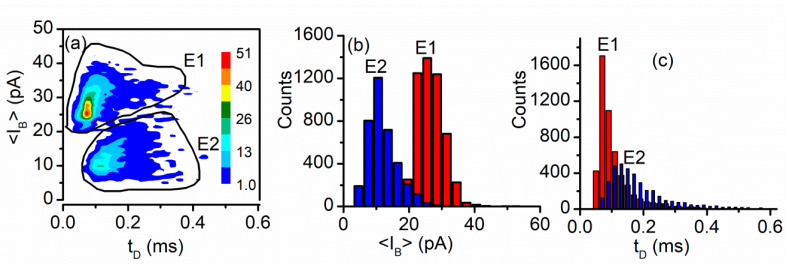
Event analysis for Ang II translocation. (**a**) The density plot shows two well-defined clusters of recorded events; the color map indicates event density; (**b**) The current blockage distribution shows a good separation between the two types of events; (**c**) The distribution in terms of dwell type between the two types of events indicates overlapping and poor separation. Published under Creative Commons Attribution License in [29].

Signal analysis in terms of the average current blockage <I_B_> and dwell time t_D_ performed with the Transalyzer software package [91] provides some important insights into the origin of recorded events. The density plot of the recorded events shows two relatively well separated clusters, E1 and E2, respectively (Figure 20a). Further analysis of the events in each cluster indicates a good separation in terms of current blockage <I_B_> (Figure 20b) and overlapping in terms of dwell time (Figure 20c). The existence of multiple clusters more or less overlapped is common for translocation experiments, especially when short peptides are used as analytes. In this case, based on previous explanations provided for similar experiments it was concluded that the events E1 are characteristic to Ang II molecules that translocated through the open channel, while E2 events represent collisions with the pore [29].

**Figure 21:**
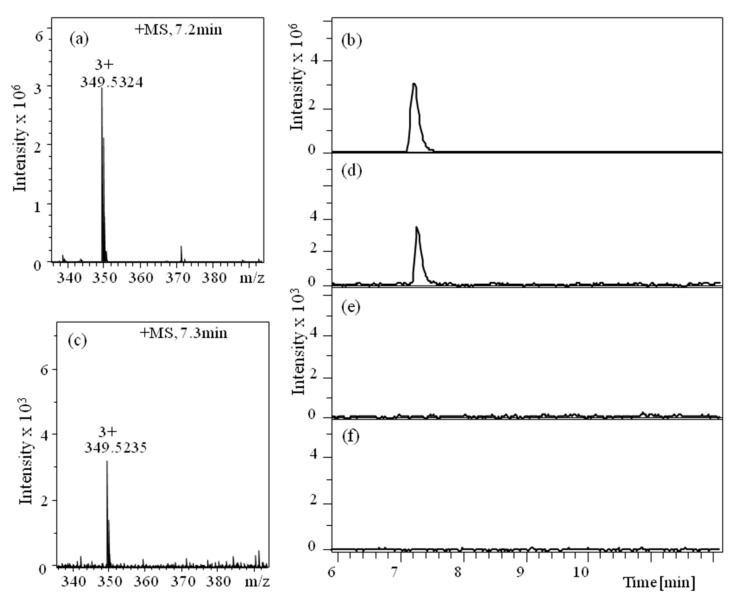
LC-MS identification of translocated Ang II peptide molecules. (**a**) MS reference indicates an m/z ratio of 349.5 (z = 3+) for a lysenin sample. (**b**) LC chromatogram of the reference Ang II. MS (**c**) and LC (**d**) detect and identify Ang II translocated into the negatively charged reservoir. No Ang II is detected upon application of positive transmembrane voltages (**e**) or following lysenin channel blockage by chitosan (**f**). Published under Creative Commons Attribution License in [29].

An important exploration of the same work provides evidence of translocation [29]. Such strong evidence is quite rare for proteins and peptides since they cannot be amplified like DNA, therefore the number of translocated molecules is very small and their detection requires very sensitive techniques to be employed [75,92]. To bring evidence of translocation, the investigators took advantage of the long-term stability presented by large populations of lysenin channels reconstituted into planar lipid membranes. Extended translocation experiments (36 h), in conjunction with large amounts of analytes and numerous channels available for translocation (over 22,000) allowed liquid chromatography—mass spectrometry (LC-MS) identification of Ang II driven by electrophoretic forces on the other side of the membrane (Figure 21) through the channels [29].

## 4. Conclusions and Perspective

Lysenin channels are molecular tools anticipated to significantly contribute to the development of high-performance sensing devices. Such devices may be realized based on the intrinsic properties of lysenin channels to adjust their conductance in response to interactions with multivalent ions. While the response is non-specific, such a simple device may find applicability for fast screening purposes. Irreversible channel blockage by cationic polymers can be realized at concentrations in the nM range [29]. Given the bio-inertness of chitosan, this particular irreversible blockage was recently exploited for temporary permeabilization of live cells and access of non-permeant molecules to the cytosol while maintaining an excellent viability of the target cells [93]. Reversible permeabilization of artificial spherical cell membranes (liposomes) was achieved by employing lysenin channels, La^3+^ ions, and EDTA [93], which may open novel avenues for drug loading into liposomal carriers and controlled release at the desired sites.

Lysenin is a protein amenable to chemical and genetic modifications intended to adjust its sensitivity and specificity for analytes. A lysenin channel inserted into an artificial lipid membrane and endowed with a biorecognition element may lead to the development of single-molecule sensors for molecules and complexes too large to thread the pore; in this case, the binding event near the pore entrance may reduce the ionic flow and facilitate electrical detection from changes in ionic currents.

## Figures and Tables

**Table 1:** Fit values of *IC*_50_ and *n* for ATP, ADP, and AMP inhibition effects on lysenin channel conductance.

	*IC*_50_ (mM, ±SD)	*n*
ATP	4.53 ± 0.07	4.15 ± 0.2
ADP	8.92 ± 0.07	3.43 ± 0.16
AMP	13.43 ± 0.08	1.62 ± 0.17

**Table 2:** Fit values of *IC*_50_ and *n* for ionic screening effects on ATP inhibition of lysenin channel conductance.

KCl (mM)	*IC*_50_ (mM, ±SD)	*n*
50	3.83 ± 0.05	4.11 ± 0.16
135	4.36 ± 0.07	4.14 ± 0.2
500	6.94 ± 0.07	4.1 ± 0.14

## Appendix A

### Experimental Details

A typical experimental setup for investigations of lysenin channels’ sensing capabilities and identification of potential mechanisms of interactions leading to adjustments of the ionic currents is depicted in Figure A1.

The setup consists of two PTFE reservoirs (each of ~1 mL volume) separated by a thin PTFE film (120 µm thickness) in which a small central hole (~70 µm diameter) is produced by an electric spark. Each reservoir is filled with an electrolyte solution, and two Ag/AgCl electrodes ensure electrical connections with the electrophysiology amplifier. Reports in the literature for similar setups are not necessarily consistent with defining each reservoir (i.e., *cis*, or *trans*), therefore the electrical connections may be used for unequivocal identification (i.e., headstage-connected and ground-connected reservoir, respectively). The bilayer is produced by the painting method, and its formation and integrity are monitored by real time capacitance and conductance measurements. Once a stable bilayer lipid membrane is achieved, channel reconstitution is performed by addition of small amounts of lysenin monomer to the grounded reservoir while applying a negative voltage to the headstage-connected reservoir (to prevent voltage induced gating). The concentration of lysenin in the reservoir varies from sub-pM to nM, and depends on the monomer source, purity, and targeted number of inserted channels. Macroscopic conductance measurements imply using a large number of channels, while investigations on regulatory mechanisms and molecule translocations require using single channels. When single channels are needed, complete exchange with monomer-free electrolyte solution in the grounded reservoir right after the first insertions may prevent reconstitution of an excessive number of channels into the membrane.

The macroscopic conductance of large populations of inserted lysenin channels before and after analyte additions is estimated from the slope of the linear IV plots recorded in response to voltage ramps within the negative voltage range; the plots may be recorded by using a low sampling rate (i.e., one sample/s) and cut-off frequency of the hardware low-pass filter. Analysis of regulatory mechanisms on single channels and macromolecule translocation experiments require using a high sampling rate (up to 2.5 × 10^5^ samples/s) and cut-off frequency to observe fast changes in ionic currents through individual channels and prevent signal alteration by excessive filtration.
